# Color, flavonoids, phenolics and antioxidants of Omani honey

**DOI:** 10.1016/j.heliyon.2018.e00874

**Published:** 2018-10-21

**Authors:** Mohamed Al-Farsi, Abeer Al-Amri, Ahlam Al-Hadhrami, Sharifa Al-Belushi

**Affiliations:** Food & Water Laboratories Center, Ministry of Regional Municipalities & Water Resources, P O Box 323, P C 100, Muscat, Oman

**Keywords:** Food science

## Abstract

Our objectives were to analyze and correlate the color, flavonoids, phenolics and antioxidants of 26 honey samples from 6 honey producing regions in the Sultanate of Oman. The Pfund method was used to measure color, aluminum chloride method for flavonoids determination, Folin-Ciocalteu method for phenolic measurement and DPPH assay to determine antioxidants. Sumer honey was the highest among other samples in terms of color, which ranged 129.8–336.2 mm pfund, flavonoids (1613–2890 mg/kg), phenolics (1624–2898 mg/kg) and antioxidants (7.8–48.6 mg/ml). There was a strong correlation between color, flavonoids and phenolics, where it's moderate between these parameters and antioxidants. The Omani honey was rich in color and phenolics compare to other honey and regarded as a good source of antioxidants to the human diet.

## 1 Introduction

Oman regard one of the main honey-producing countries in the Arabian Peninsula with production in the year 2016 reached 600 metric tons (Annual Report, 2017; Al-Farsi et al., 2018). Honey is part of the human diet since ancient times which produced by several species of honeybee *Apis mellifera*. It is regarded as the main product of the beekeeping sector, both from the quantity and economic points of view (Krell, 1996). In Oman there are two main florals for honey, the *Acacia tortilis* which locally called Sumer, and the *Ziziphus spina-Christi*, which called Sidr (Sajwani et al., 2007; Al-Farsi et al., 2018). The honey composition can vary significantly depending on the bee species, region, source of nectar and mode of harvesting and post-harvest condition (Singhal et al., 1997). Honey composed mainly of sugars which ranged between 70 and 85%, where fructose and glucose are the major sugars, and water is the second largest component in honey (15–20%) (Krell, 1996). Honey also contains other minor components such as proteins, enzymes, minerals, vitamins, organic acids and phenolics (Roshan et al., 2017). Even when honey is made from the same floral and geographical origin, honey can vary in texture, color, and composition depending on the bee species, soil, weather conditions, and even the age of the bees, which greatly affects the enzymatic activity that produces the honey (Sant Ana et al., 2014).

Honey is highly regarded for its nutritional value, healing properties and has been used in traditional medicine in many countries. The functional and biological properties of honey proved by several studies (Estevinho et al., 2008; Gheldof et al., 2002; Irish et al., 2008), such as antioxidant, anti-inflammatory, antibacterial, antiviral, antiulcerative, antilipid and anticancer properties. These activities mainly attributed to the phenolic compounds in honey, such as the flavonoids, which have antioxidant properties and radical-scavenging activities (Khalil et al., 2012).

Antioxidants are substances that protect the cells of the body from damage caused by unstable molecules known as free radicals. The antioxidants present in honey include both enzymatic substances (e.g. glucose oxidase, catalase and peroxidase) and non-enzymatic substances, such as ascorbic acid tocopherol, carotenoids and more than 150 polyphenolic compounds, including flavonoids, flavonols, phenolic acids, catechins and cinnamic acid derivatives (AlMamary et al., 2002; Khalil et al., 2012). Plant polyphenols are primary natural antioxidants having many beneficial functions such as reducing agents, metal chelators, and singlet oxygen quenchers (Duthie et al., 2000). Flavonoids and simple phenolic derivatives such as phenolic acids are representing the majority of plant polyphenols and considered as the main antioxidants in honey (Bravo, 1998). The average antioxidant contents of honey have been determined and found to be correlated to their phenolic contents (Gheldof et al., 2002). Polyphenol has also been reported to affect the flavor (Steeg and Montage, 1988) and physical appearance of honey, particularly honey color (Alvarez-Suarez et al., 2010; Isla et al., 2011). Bertoncelj et al. (2007) reported a positive correlation between phenolic content, color, and antioxidant activity in honey.

In monofloral honey, flavonoids are major components, which are up to 42% of total phenolics (Sabatier et al., 1992). However, flavonoids profiles in honey generally vary widely and they are mainly different according to floral and geographical origins (Soler et al., 1995). Also, Maurya et al. (2014) stated that the botanical origin of honey has the greatest influence on its antioxidants and phenolics while processing handling and storage affect honey on a minor degree.

Many methods for determining the antioxidant activity of honey have been used, an assay based on the use of DPPH radicals are among the most popular spectrophotometric methods (Bendini et al., 2006). The DPPH scavenging method is preferred for its simple, rapid, sensitive and reproducible procedure, also because of the chromogenic radical compounds (DPPH) can directly react with antioxidants (Ozcelik et al., 2003). Although several studies worldwide were performed on the antioxidants activities of honey, no studies reported the antioxidants in Omani honey. Therefore, the aims of this study were to quantitatively analyze the color intensity, phenolic, flavonoid, and antioxidant properties of honey samples collected from different regions in Oman. Also to describe the relationship between these parameters using Pearson's correlation and determined how these parameters can differentiate between various honey types.

## 2 Materials & methods

### 2.1 Honey samples

The honey samples of *Apis mellifera* honeybee, investigated in this study, were collected in December 2016 from 6 regions in the Sultanate of Oman. They were 26 samples in total; 10 Sumer, 10 Sidr and 6 multiflora. Table 1 shows common names, floral origin and geographical origin of these samples. The samples were stored at room temperature in a dark place until analyses.

**Table 1:** Characterization of honey samples.

Sample code	Common name	Floral origin	Geographical origin
SM 1	Sumer	*Acacia tortilis*	Dema w Thaieen/Northeastern
SM 3	Sumer	*Acacia tortilis*	Al Awabi/Batinah South
SM 6	Sumer	*Acacia tortilis*	Al Hawqayn/Batinah South
SM 7	Sumer	*Acacia tortilis*	Mahdah/Buraimi
SM 10	Sumer	*Acacia tortilis*	Khaboura/Batinah North
SM 14	Sumer	*Acacia tortilis*	Ibri/Dhahirah
SM 23	Sumer	*Acacia tortilis*	Shinas/Batinah North
SM 31	Sumer	*Acacia tortilis*	Khaboura/Batinah North
SM 46	Sumer	*Acacia tortilis*	Samail/Interior
SM 57	Sumer	*Acacia tortilis*	Rustaq/Batinah South
SD 2	Sidr	*Ziziphus spina-christi*	Dema w Thaieen/Northeastern
SD 4	Sidr	*Ziziphus spina-christi*	Al Awabi/Batinah South
SD 5	Sidr	*Ziziphus spina-christi*	Hawqayn/Batinah South
SD 8	Sidr	*Ziziphus spina-christi*	Mahdah/Buraimi
SD 9	Sidr	*Ziziphus spina-christi*	Khaboura/Batinah North
SD 15	Sidr	*Ziziphus spina-christi*	Nizwa/Interior
SD 17	Sidr	*Ziziphus spina-christi*	Bani Khalid/Northeastern
SD 24	Sidr	*Ziziphus spina-christi*	Shinas/Batinah North
SD 27	Sidr	*Ziziphus spina-christi*	Sohar/Batinah North
SD 58	Sidr	*Ziziphus spina-christi*	Ibra/Northeastern
FL 25	Flower	Multiflora	Shinas/Batinah North
FL 29	Flower	Multiflora	Rustaq/Batinah South
FL 38	Flower	Multiflora	Samail/Interior
FL 39	Flower	Multiflora	Izki/Interior
FL 54	Flower	Multiflora	Ibra/Northeastern
FL 55	Flower	Multiflora	Ibra/Northeastern

### 2.2 Color intensity

Color intensity was determined according to Ferreira et al. (2009) and Lacerda et al. (2010). Honey samples were diluted to 50% with distilled water, mixed, and centrifuged at 3200 rpm/5 min. The absorbance was measured at 635 nm using a spectrophotometer (Thermo Sci., Evolution 201, China), and color intensity was determined using the Pfund scale using the following equation.

Pfund = −38.70 + 371.39 × Abs

### 2.3 Total flavonoids

Total flavonoids content in honey was determined by a calorimetric method according to Zhishen et al. (1999). Briefly, one ml of diluted honey (0.2 g/ml) was mixed with 4 ml of distilled water followed by 0.3 ml of 5% sodium nitrite. After 5 min, 0.3 ml of 10% aluminum chloride was added, six min later 2 ml of 1M of sodium hydroxide was added and the volume was increased to 10 ml by distilled water. The mixture was shaken and absorbance was measured at 510 nm using a spectrophotometer. A calibration curve was made using a standard solution of catechin 20–100 mg/l. The results were presented as mg of catechin equivalents per kg of honey.

### 2.4 Total phenolics

The total phenolic content was measured using the Folin-Ciocalteu method according to Socha et al. (2009) and Ferreira et al. (2009). The honey stock solutions were prepared at a concentration of 0.04 g/mL of distilled water. A portion of 0.5 mL of the stock solution was mixed with 0.3 mL of Folin-Ciocalteu reagent and followed by 2 mL of 15% sodium carbonate. Distilled water was added to 5 ml and the mixture mixed. The mixture incubated for 2 hrs, and the absorbance of the mixture was measured at 798 nm. A standard curve of gallic acid was prepared for quantification, using a concentration range between 8 and 40 mg/l and the results were expressed as mg gallic acid/kg honey.

### 2.5 Antioxidants DPPH assay

DPPH assay was estimated using the 2,2-diphenyl-1-picrylhydrazyl hydrate radical (DPPH) according to Hatano et al. (1988). The honey samples were diluted in distilled water at concentrations from .05 to .25 g/mL, and from each dilution 0.3 ml was mixed with 2.7 mL of DPPH (0.002 g/100 ml methanol). The mixtures were vortexed, left in dark room temperature for 60 min and the absorbance was measured at 517 nm. The Radical Scavenging Activity (RSA) was calculated as a percentage of DPPH using the following equation:

% RSA = (Abs. DPPH − Abs. sample/Abs. DPPH) × 100

The 50% of RSA (EC50) was calculated by interpolation from the graph of % RSA against sample concentration.

### 2.6 Statistical analysis

The results were reported as a mean ± standard deviation from the triplicate analysis. Pearson's correlation coefficient was measured to find the association between two variables of color, flavonoids, phenolic and antioxidants, using Microsoft Excel.

## 3 Results and discussion

Tables 2, 3 and 4 presented color (mm Pfund), flavonoids (mg/kg), phenolics (mg/kg) and antioxidants (IC50 mg/ml) in Sumer, Sidr and multiflora honeys respectively.

**Table 2:** Color, flavonoids, phenolics and antioxidants activity of Sumer samples.

Sample code	Color (mmPfund)	Color	Flavonoids (mg/kg)	Phenolics (mg/kg)	Antioxidants (IC50 mg/ml)
SM 1	156.2 ± 1.2	Dark amber	1666 ± 40	1718 ± 88	31.3
SM 3	285.3 ± 3.5	Dark amber	2480 ± 39	2698 ± 83	17.6
SM 6	129.8 ± 0.9	Dark amber	2140 ± 30	2146 ± 20	26.3
SM 7	211.8 ± 0.2	Dark amber	2314 ± 51	2403 ± 55	17.3
SM 10	230.4 ± 0.7	Dark amber	1912 ± 33	2025 ± 75	32.7
SM 14	248.9 ± 5.2	Dark amber	1613 ± 29	1624 ± 22	7.8
SM 23	336.2 ± 0.2	Dark amber	2890 ± 36	2898 ± 19	23.4
SM 31	139.9 ± 0.2	Dark amber	1799 ± 11	2178 ± 102	25.6
SM 46	269.9 ± 0.7	Dark amber	2316 ± 14	2322 ± 30	20.7
SM 57	262.3 ± 1.7	Dark amber	2301 ± 15	2345 ± 9	48.6
Average	227.1	Dark amber	2143	2236	25.1

The values are mean of triplicate ± standard deviation.

**Table 3:** Color, flavonoids, phenolics and antioxidants activity of Sidr samples.

Sample code	Color (mmPfund)	Color	Flavonoids (mg/kg)	Phenolics (mg/kg)	Antioxidants (IC50 mg/ml)
SD 2	135.1 ± 0.1	Dark amber	993 ± 37	1123 ± 59	41.5
SD 4	94.2 ± 0.1	Amber	696 ± 9	1081 ± 61	72.3
SD 5	97.6 ± 0.2	Amber	884 ± 8	1447 ± 91	45.5
SD 8	106.1 ± 2.4	Amber	987 ± 31	1520 ± 12	41.2
SD 9	122.3 ± 0.7	Dark amber	685 ± 1	1015 ± 39	57.2
SD 15	119.9 ± 0.7	Dark amber	1034 ± 35	1515 ± 54	58.1
SD 17	92.7 ± 0.2	Amber	635 ± 1	972 ± 26	41.5
SD 24	117.1 ± 0.9	Dark amber	944 ± 2	1353 ± 17	64.0
SD 27	87.8 ± 0.5	Amber	718 ± 2	1302 ± 56	33.8
SD 58	121.9 ± 8	Dark amber	899 ± 42	1375 ± 11	38.2
Average	109.5		848	1270	49.3

The values are mean of triplicate ± standard deviation.

**Table 4:** Color, flavonoids, phenolics and antioxidants activity of multiflora samples.

Sample code	Color (mmPfund)	Color	Flavonoids (mg/kg)	Phenolics (mg/kg)	Antioxidants (IC50 mg/ml)
FL 25	95.2 ± 0.5	Amber	787 ± 14	842 ± 6	190.1
FL 29	65.5 ± 0.5	Light amber	521 ± 6	949 ± 38	176.5
FL 38	106.6 ± 3.1	Amber	883 ± 28	1208 ± 19	97.5
FL 39	180.5 ± 1.7	Dark amber	1354 ± 13	1384 ± 13	91.2
FL 54	111.5 ± 0.5	Amber	993 ± 49	998 ± 40	160.9
FL 55	147.6 ± 0.7	Dark amber	1011 ± 14	1015 ± 9	150.8
Average	117.8		925	1066	144.5

The values are mean of triplicate ± standard deviation.

### 3.1 Color

Color values presented in Pfund values (mm) and Pfund scale (water white, extra white, white, extra light amber, light amber, amber and dark amber) in order to classify honey colors. Sumer samples ranged between 129.8 and 336.2 mm which is equivalent to dark amber for all Sumer samples. Whereas Sidr honey ranged between 87.8 and 135.1 mm which represent color between amber and dark amber and multiflora honey was ranged between 65.5 and 180.5 mm which is between light amber and dark amber. The Sumer (Acacia) honey was the darker honey compare to Sidr and multiflora honey. Moniruzzaman et al. (2013) reported lower color values for Malaysian Acacia, their value ranged between 82 and 150 mm. Our Sidr and multifloral color were similar to values reported for Algerian honey which ranged between 31 and 198 mm (Rebia and Lanez, 2014), Brazilian honey which ranged between 31 and 166 mm (Pontis et al., 2014) and for Argentinian honey color ranged between 40.7 and 140 mm (Cabrera et al., 2017).

Color is a sensory attribute impotent to consumer preference of honey quality. The international markets demand specific honey colors, for example, Europe prefer darker honey with potent flavors while in North America prefer light color honey with less intense flavor (Delmoro et al., 2010). The color of each honey is due to pigments such as carotenoids and flavonoids which is dependent on the botanical and geographical origin of the product (Terrab et al., 2003). Aging and storage conditions may also affect the color intensity. The color and consistency of honey depend also on the content of water, saccharides and pollens (Baltrusaityte et al., 2007). Several studies show that dark honey have higher values of phenolic content and antioxidants than lighter honey (Ferreira et al., 2009; Alvarez-Suarez et al., 2010; Perna et al., 2013; Cabrera et al., 2017).

### 3.2 Total flavonoids

Flavonoids are polyphenol plant pigments that are synthesized from the amino acid phenyl alanine. They contain several subclasses including anthocyanin, catechins, flavanone glycosides, flavanone, flavons, flavonol glycosides, flavonols and isoflavon (Hamdy et al., 2009). Flavonoids in honey may originate from nectar, pollen or propolis (Hamdy et al., 2009). The flavonoid content was measured using a colorimetric method, which based on the formation of a complex between the aluminum ion and the carbonyl and hydroxyl groups of flavonoids that produce a yellow color. The total flavonoids in our samples were highest in Sumer samples which ranged between 1613 and 2890 mg/kg, while in Sidr samples ranged between 635 and 1034 mg/kg and between 521 and 1354 mg/kg in multiflora samples. These results were higher than values reported for Argentinian honey 69.4–677.6 mg/kg (Cabrera et al., 2017); Portuguese honey 123.6–587.4 mg/kg (Ferreira et al., 2009); Moroccan honey 11.7–179.1 mg/kg (Bouhlali et al., 2016) and Malaysian honey 135.3–165.3 mg/kg (Khalil et al., 2012). The higher flavonoids in our samples compare to others could be due to botanical and geographical differences as well to climate and environmental factors such as humidity, temperature and soil composition.

### 3.3 Total phenolics

Polyphenols constitute one of the most numerous and widely distributed groups of substance in the plant kingdom, whereas flavonoids and simple phenolics derivatives were the most common polyphenols (Cabrera et al., 2017). A referenced colorimetric method was used to measure total phenolics in our samples, using Folin-Ciocalteu as the reagent. The reaction between Folin-Ciocalteu and phenolic compounds results in formation of blue color which allow quantification using gallic acid as standard.

The total content of phenolics was ranged between 1624 and 2898 mg/kg for Sumer honey, 972–1520 mg/kg for Sidr honey and 842–1384 mg/kg for multiflora honey. The phenolic content in Sumer honey was much higher than other samples. Generally our samples show higher phenolic content compared to honey from other countries; Malaysian honey 305.5–419.9 mg/kg (Khalil et al., 2012); Czech honey 39.2–167.1 mg/kg (Lachman et al., 2010); Portuguese honey 132.2–727.8 mg/kg (Ferreira et al., 2009) and Brazil 250–548 mg/kg (Pontis et al., 2014). The most similar phenolic contents to Omani honey was reported for Mexican honey 621.3–1368.4 mg/kg (Tapia-Campos et al., 2017); Moroccan honey 239.5–1138.5 mg/kg (Bouhlali et al., 2016) and Argentinian honey 401.8–1188.2 mg/kg (Cabrera et al., 2017). The high phenolic content in the investigated Omani honey confirmed the good quality of the honey. The variation in the phenolic content for our samples compared to those from different countries may be due to the different geographical and botanical sources of honey.

### 3.4 Total antioxidants

DPPH method was chosen to measure antioxidants in honey because it's easy, precise and accurate method (Pontis et al., 2014). The evaluation of antioxidants was determined based on the scavenging activity against the free radical DPPH through the IC50 parameter, which represents the concentration of the material necessary to inhibit 50% of free radical. Thus a lower IC50 value in honey indicates a greater antioxidants and ability to neutralize free radicals. The antioxidants values of our samples ranged between 7.8 and 48.6 mg/ml for Sumer samples, 33.8–72.3 mg/ml for Sidr samples and 91.2–190.1 mg/ml for multiflora samples. Sumer honey was the highest in antioxidants among other samples which indicated by their darker color and higher phenolics contents. Similar results reported in the literature, a review study for Maurya et al. (2014) reported the antioxidants (IC50) in Acacia from different countries, their values ranged between 10.5 and 111.1 mg/ml. Also reported antioxidants in multiflora honey which ranged between 4.4 mg/ml in Croatian honey to 358 mg/ml in honey from Czech. The IC50 for Portuguese honey was 84.9–168.9 mg/ml (Ferreira et al., 2009) and 5.9–89.7 mg/ml in Iranian honey (Tahmasbi et al., 2015).

Several types of compounds can contribute to the antioxidants in honey including carotenoids, ascorbic acid, tocopherols and polyphenol compounds. The variation in antioxidants among unifloral honey, with different geographical origin may be due to climate and environmental factors such as temperature, humidity and soil composition, as well as post-harvest condition.

### 3.5 Correlation

Correlation established between color, flavonoids, phenolics and antioxidants presented in Table 5 to explore the possible relationships between these values. There was a strong positive correlation between color: flavonoids (0.999) and color: phenolics (0.974), as well as between flavonoids: phenolics (0.977). Whereas the relation was negative between antioxidants: color (−0.608), antioxidants: flavonoids (−0.616) and antioxidants: phenolics (−0.771). This negative relation between antioxidants and other parameters due to IC50 calculation, which low value indicates greater antioxidants.

**Table 5:** Correlation established between color, flavonoids, phenolics and antioxidants.

	Color	Flavonoids	Phenolics	Antioxidants
Color	1			
Flavonoids	0.999	1		
Phenolics	0.974	0.977	1	
Antioxidants	−0.608	−0.616	−0.771	1

The strongest correlation existed between color and both flavonoids and phenolics indicating that the darker honey can be attributed to the presence of high amount of flavonoids and phenolics which increase the amount of antioxidants. For instance, the average value of color was the highest in Sumer honey (227.1 mm) among other samples, which indication for higher flavonoids (2143 mg/kg), higher phenolics (2236 mg/kg) and higher antioxidants (25.1 mg/ml). The strongest correlation between antioxidants and phenolics suggest that phenolics are the strongest contributor to the radical scavenging activity in honey samples. A similar degree of correlations has been demonstrated by several studies. Pontis et al. (2014) reported color: phenolics correlation (.967), color: antioxidants (−0.800) and phenolics: antioxidants (−0.752). Moniruzzaman et al., 2013 reported correlation for Acacia honey, color: phenolics (0.816), color: antioxidants (0.820) and phenolics: antioxidants (0.785). Bouhlali et al. (2016) reported a strong correlation between antioxidants: phenolics (0.962), and antioxidants: flavonoids (0.847).

## 4 Conclusions

This is the first study classifying color, flavonoids, phenolics and antioxidants properties of Omani honey. These parameters varied in different types of Omani honey, where Sumer honey was the richest in color, flavonoids, phenolics and antioxidants among other samples. A strong correlation was observed between the color, flavonoids, phenolics and antioxidants of honey. We can conclude that Sumer honey was the best among the analyzed samples in terms of high color, phenolics and antioxidants. The studied Omani honey, compared to honey from other countries, were rich in phenolics and showed a noticeable antioxidants activity, these are an interesting feature to take into account in human health. Therefore honey can be used as a natural food ingredient as well as a rich source of antioxidants in the human diet.

## Declarations

### Author contribution statement

Mohamed Al-Farsi: Conceived and designed the experiments; Analyzed and interpreted the data; Contributed reagents, materials, analysis tools or data; Wrote the paper.

Abeer Al-Amri, Ahlam Al-Hadhrami, Sharifa Al-Belushi: Performed the experiments; Contributed reagents, materials, analysis tools or data.

### Funding statement

This work was supported by Food & Water Laboratories Center.

### Competing interest statement

The authors declare no conflict of interest.

### Additional information

No additional information is available for this paper.
